# Spatial Transcriptomics in Lung Cancer and Pulmonary Diseases: A Comprehensive Review

**DOI:** 10.3390/cancers17121912

**Published:** 2025-06-09

**Authors:** Da Hyun Kang, Yoonjoo Kim, Ji Hyeon Lee, Hyeong Seok Kang, Chaeuk Chung

**Affiliations:** Division of Pulmonology and Critical Care Medicine, Department of Internal Medicine, College of Medicine, Chungnam National University, Daejeon 34134, Republic of Korea; ibelieveu113@cnuh.co.kr (D.H.K.); too9655@naver.com (J.H.L.); wooayyak@daum.net (H.S.K.)

**Keywords:** spatial transcriptomics, lung cancer, tumor microenvironment, chronic obstructive pulmonary disease, idiopathic pulmonary fibrosis, asthma, immunotherapy, fibroblasts, biomarker discovery, single-cell resolution

## Abstract

Recent advancements in spatial transcriptomics (ST) have revolutionized our understanding of the lung’s cellular organization and pathological alterations. By preserving the spatial distribution of gene expression, ST reveals localized immune niches, stromal–epithelial interactions, and disease-associated transcriptional “hotspots” that cannot be captured by conventional sequencing methods alone. In lung cancer, ST-based investigations have delineated distinct tumor microenvironments between tumor cores and invasive fronts, revealing prognostically significant gene signatures and identifying subpopulations with differential responses to immunotherapy and chemotherapy. Similarly, in chronic obstructive pulmonary disease, asthma, and idiopathic pulmonary fibrosis, ST has mapped the ecosystem, including immune cells, inflammatory mediators, and fibroblast subtypes, of discrete regions within diseased lung tissue, offering mechanistic insights into disease progression and tissue remodeling. In addition, a more recent ST study provides spatial information for where drugs act within tissues. This review highlights the emerging role of spatial transcriptomics in respiratory research, demonstrating its potential to refine disease classification, elucidate mechanisms of therapeutic resistance, and inform spatially guided personalized interventions in respiratory diseases.

## Simple Summary

Spatial transcriptomics (ST) is an innovative technology that maps gene expression while preserving tissue structure, offering new perspectives on disease mechanisms in the lung. This review explores how ST has advanced our understanding of lung cancer and other pulmonary diseases. In lung cancer, ST helps reveal tumor heterogeneity, immune evasion mechanisms, and spatially distinct cellular subtypes that influence therapy response. In diseases such as pulmonary fibrosis, COPD, and asthma, ST identifies localized immune niches, fibroblast diversity, and vascular remodeling that drive disease progression. The review also compares major ST platforms like GeoMx, Visium, and Xenium and highlights current clinical trials and future directions. By integrating molecular insights with spatial context, ST holds great promise for improving diagnosis, treatment, and precision medicine in respiratory diseases.

## 1. Introduction

Spatial transcriptomics (ST) is an emerging set of techniques that allow researchers to visualize and quantify gene expression in situ, preserving the spatial architecture of the tissue [1,2,3]. Unlike bulk RNA sequencing (RNA-seq) or single-cell RNA sequencing (scRNA-seq), which typically lose or disrupt spatial information, ST methods maintain the tissue’s morphological context, making it possible to pinpoint exactly where certain transcripts are expressed within a tissue section [4,5]. ST enables the profiling of gene expression across distinct tissue regions, providing critical insights into intratumoral heterogeneity and the molecular characteristics of localized cellular microenvironments [6,7]. This spatially resolved transcriptomic data can provide unique insights into cellular organization, cell–cell interactions, and disease pathology [8].

### 1.1. Research Methodology for Article Selection

To ensure a comprehensive and up-to-date synthesis of current advancements in ST as applied to lung cancer and pulmonary diseases, we conducted a structured literature review using major biomedical databases, including PubMed, Web of Science, and Scopus. Searches were performed using combinations of the following keywords: “spatial transcriptomics”, “lung cancer”, “pulmonary fibrosis”, “Chronic obstructive pulmonary disease (COPD)”, “asthma”, “lung adenocarcinoma”, “tumor microenvironment”, and “immune landscape.” Articles published between January 2018 and May 2025 were considered, with a focus on peer-reviewed original research articles, clinical studies, and high-impact reviews that employed spatially resolved transcriptomic techniques.

Inclusion criteria were as follows: studies employing spatial transcriptomics platforms (e.g., Visium (10x Genomics, Pleasanton, CA, USA), Xenium (10x Genomics, Pleasanton, CA, USA), GeoMx Digital spatial profiler (DSP) (Nanostring Technologies, Seattle, WA, USA) applied to lung or pulmonary tissues; original research or clinical data with spatially annotated transcriptomic outputs; articles that provided mechanistic insights, clinical correlations, or methodological innovations relevant to respiratory disease; English-language publications with available full texts.

Exclusion criteria included commentaries, conference abstracts without full data, and studies not directly involving pulmonary diseases or spatial transcriptomic methodologies. Selected studies were independently reviewed by the authors for relevance, methodological rigor, and contribution to the understanding of spatial heterogeneity, disease progression, or therapeutic response. References were cross-validated for consistency and accuracy using DOI and PubMed identifiers, ensuring inclusion of both foundational studies and the latest research.

### 1.2. Historical Perspective of Spatial Transcriptomics

Over the past two decades, technological advances in Next-Generation Sequencing (NGS) have transformed genomic research by enabling high-throughput characterization of DNA and RNA. This era saw bulk RNA-seq become a standard approach for analyzing transcriptomes at scale; however, because it requires homogenized tissues, this technique averages signals across heterogeneous cell populations and obscures the cellular diversity and spatial arrangements within a sample [9].

In response to these limitations, scRNA-seq emerged, offering transcriptomic resolution at the level of individual cells. By isolating single cells, scRNA-seq captures rare or specialized populations and highlights cell-specific gene signatures and pathways. Nonetheless, the dissociation process often disrupts native tissue architecture crucial for understanding how cells interact within complex tissues, and thus, scRNA-seq typically lacks spatial context [10,11].

The recognition that spatial organization was essential for understanding many biological processes led to the development of ST, which seeks to preserve tissue morphology while also capturing quantitative gene expression [5,12]. Early ST methods typically used tissue sections placed onto slides or arrays embedded with unique molecular barcodes. When the tissue is permeabilized, messenger RNA (mRNA) transcripts diffuse out into these barcoded capture zones, allowing the transcripts’ origin to be mapped back to specific locations on the tissue section [13]. By sequencing the captured cDNA and retaining knowledge of which spatial barcode was associated with each read, investigators obtain a transcriptome-wide dataset linked to discrete tissue coordinates [1,3,14].

Whole Exome Sequencing (WES) focuses on protein-coding regions, which make up only a small fraction of the genome. By selectively capturing exons before sequencing, WES offers a cost-effective way to identify disease-causing variants or driver mutations in cancer. However, it cannot detect noncoding variants or measure gene expression, potentially missing important regulatory regions and epigenetic factors [15,16,17].

Whole Genome Sequencing (WGS) examines the entire genome, providing a comprehensive view of all variants (including single-nucleotide variants, structural variants, and noncoding alterations). As the most complete DNA-based strategy, WGS is valuable for identifying complex or large-scale genomic rearrangements and for studying the overall landscape of genomic variation. However, it demands higher costs, large data storage, and more complex analysis compared to WES [18,19].

Bulk RNA-seq, in contrast, measures mRNA expression levels across a pooled population of cells within a sample. It is widely used for differential expression analysis and biomarker discovery due to its relatively straightforward workflows and moderate cost. Nevertheless, bulk RNA-seq averages out the contributions of individual cells, obscuring any heterogeneity and making it challenging to detect subtle or rare subpopulations [10,11,20].

scRNA-seq addresses these limitations by capturing transcriptomes at the resolution of individual cells. This enables comprehensive profiling of cellular heterogeneity, discovery of rare cell types, and the reconstruction of developmental or differentiation trajectories. However, scRNA-seq data do not inherently provide spatial information, and tissue dissociation procedures can alter transcriptional states or lose certain populations. Moreover, scRNA-seq requires more specialized techniques, resulting in higher costs and more complex data analysis than bulk RNA-seq [11,20,21,22].

ST preserves the native architecture of tissue sections while capturing gene expression patterns. Depending on the platform, ST can measure whole-transcriptome or targeted gene sets within defined locations (spots, beads, or single cells), facilitating direct insights into local cell–cell interactions and tissue microenvironments. This capability is especially powerful for mapping immune niches, studying tumor heterogeneity, or investigating organ development. Despite its potential, ST remains relatively high in cost, involves sophisticated instrumentation, and yields complex datasets that demand advanced bioinformatics approaches. Furthermore, it has comparatively lower throughput than bulk RNA-seq or scRNA-seq, making it an emerging yet invaluable technique for studying spatially regulated biological processes [2,23,24,25,26].

In summary, WES and WGS primarily serve as DNA-based methods to uncover genetic variants. While WES offers a more focused approach on exons, WGS captures noncoding regions, detecting structural and copy number variations more accurately, and enabling analysis of the mitochondrial genome. WGS also improves variant calling in GC-rich or repetitive regions and identifies regulatory or intronic variants missed by WES. Bulk RNA-seq provides an averaged transcriptome view that, while cost-effective and simpler, overlooks cellular heterogeneity. Single-cell RNA-seq alleviates this shortcoming by resolving individual cell transcriptomes but loses spatial context. Spatial transcriptomics adds architectural insights by revealing how cellular gene expression patterns and interactions are organized in situ. All of these approaches are inherently complementary, and their integrative application is driving a paradigm shift in multi-omics research. By enabling the simultaneous interrogation of genomic, transcriptomic, epigenomic, proteomic, and spatial data, multi-omics is unlocking unprecedented insights into cellular heterogeneity, regulatory networks, and disease mechanisms—particularly in complex systems, such as developmental biology and precision oncology [27,28,29,30] (Table 1).

## 2. Major Platforms and Methodologies in Spatial Transcriptomics

NanoString’s GeoMx Digital Spatial Profiler (DSP) implements a “region of interest” (ROI)-based strategy that integrates photocleavable oligonucleotide tags with either RNA probes or antibody-based protein detection. In practice, researchers select specific tissue areas—often guided by histopathology, immunohistochemistry, or fluorescent markers—and illuminate those ROIs with UV light, causing the release of the oligonucleotide tags. These tags are then collected and quantified via next-generation sequencing or NanoString’s nCounter system, generating expression or protein abundance profiles that correspond to the designated areas [31,32]. One of the major strengths of GeoMx is its high multiplexing capacity, which accommodates panels of up to 18,000 RNA probes or more than 100 protein markers [33]. This platform is also well-known for its formalin-fixed paraffin-embedded (FFPE) compatibility, making archival or clinical samples accessible for ST studies. However, because the UV illumination is typically carried out over relatively broad ROIs (for example, hundreds of micrometers wide), resolution usually remains at a multi-cell level. Very small ROIs can sometimes approach near single-cell resolution, but this tends to increase the risk of low signal and technical complexity [31,34]. Additionally, the requirement for specialized laser hardware and user expertise in ROI selection can pose practical hurdles (Table 2).

In contrast, the Xenium In Situ platform from 10x Genomics detects transcripts directly within tissue sections using highly specific, barcoded probes and in situ hybridization chemistry [35]. Once hybridized and enzymatically amplified, each RNA molecule is labeled with fluorescent signals that can be imaged at single-molecule resolution. This approach grants relatively high spatial precision and strong sensitivity, enabling the localization of transcripts down to subcellular or single-cell scales. Another advantage lies in Xenium’s tight integration with the 10x Genomics ecosystem; researchers working with single-cell RNA-seq (scRNA-seq) or immune profiling solutions can leverage existing pipelines and data integration tools to correlate single-cell gene signatures with spatial expression patterns. Despite this seamless compatibility, the cost and throughput might be limiting for larger cohorts, and tissue samples must be compatible with the in situ hybridization protocols—this includes considerations for tissue thickness, autofluorescence, and the ability of probes to penetrate the tissue [5,12,20,36,37,38,39] (Table 2).

The Visium platform, also developed by 10x Genomics, combines histological imaging with spatially barcoded capture spots on a glass slide. Each spot contains unique oligonucleotide barcodes designed to bind polyadenylated mRNAs from the tissue section placed on top. Following tissue permeabilization and reverse transcription, the labeled transcripts are sequenced, yielding gene expression counts for each spot. The standard Visium approach employs spot diameters typically between 55 and 100 μm, capturing multiple cells per spot. This multi-cell resolution often provides a good compromise between cost, throughput, and ease of data analysis, though it lacks true single-cell resolution. Strengths include relatively straightforward integration with H&E staining, robust community support, and well-established computational pipelines. Additionally, there is an FFPE-compatible version of Visium that relies on targeted probes rather than oligo-dT capture, allowing formalin-fixed samples to be profiled. However, all Visium assays usually detect only polyadenylated RNA transcripts, excluding non-polyadenylated species such as long noncoding or circular RNAs [1,5,20,36,37,40] (Table 2).

### Summary and Comparative Insights

**Figure 1:**
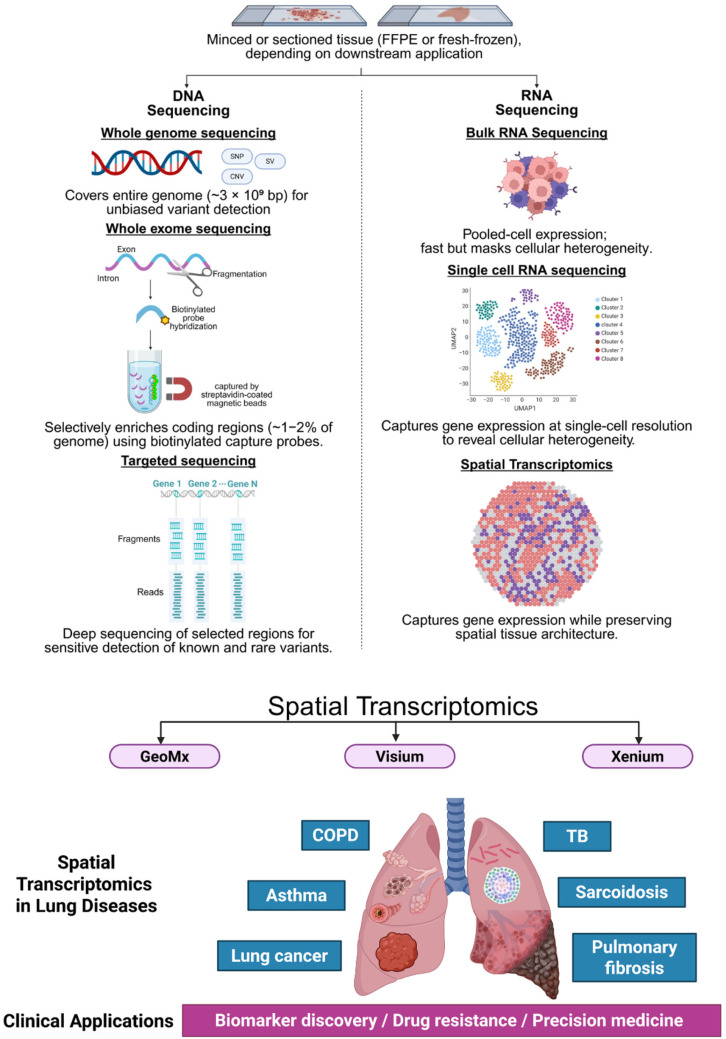
Evolution of transcriptomic technologies and applications of spatial transcriptomics in lung diseases.

**Figure 2:**
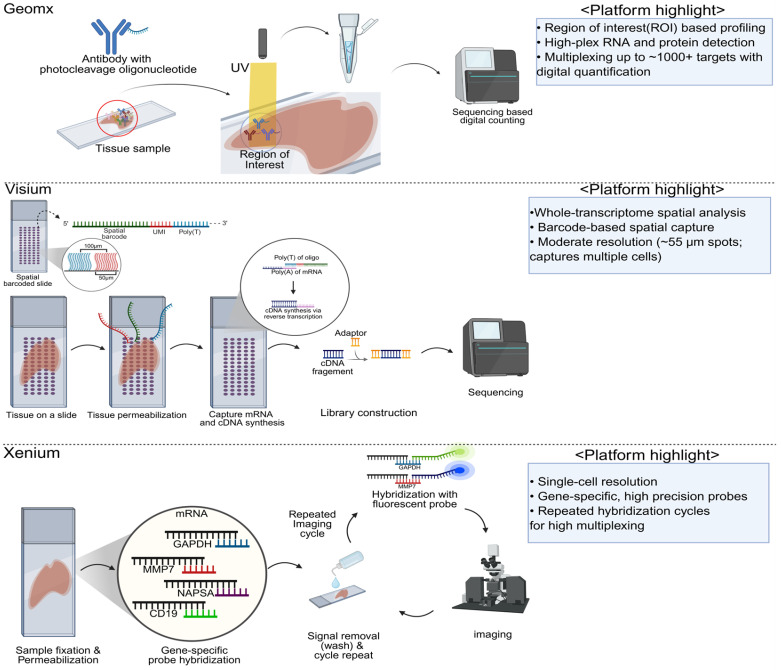
Technical principles and comparative overview of spatial transcriptomics platforms.

**Figure 3:**
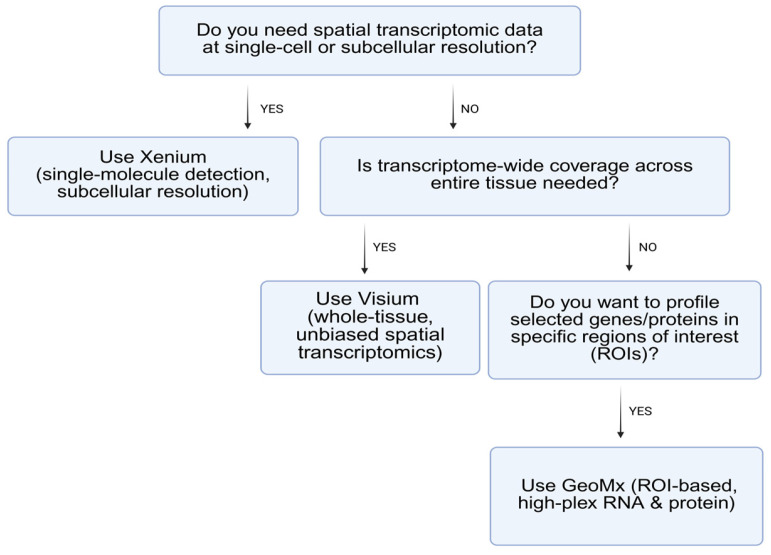
Workflow for selecting appropriate spatial transcriptomics platforms.

Spatial transcriptomics platform selection should reflect study goals. GeoMx may be ideal for targeted, high-multiplex protein+RNA quantification across specific regions, while Xenium provides high-resolution imaging of transcripts at or near single-cell resolution. Visium offers an accessible entry point into ST, albeit with lower spatial resolution than Xenium. Discovery-oriented platforms (e.g., Visium) provide broad coverage at cellular resolution, ideal for screening [35]. Validation platforms (e.g., Xenium) offer subcellular resolutions suitable for precise, targeted analysis. Resolution and transcript depth must be balanced accordingly [2,36,37,40,41]. Researchers should consider tissue compatibility, cost, throughput, and the level of spatial resolution required when selecting a platform [41,42] (Figure 1, Figure 2 and Figure 3).

This figure summarizes the progression of transcriptomic technologies from DNA-based sequencing (WES, WGS) to RNA-based bulk and single-cell RNA sequencing (bulk RNA-seq, scRNA-seq), advancing toward spatial transcriptomics. Spatial transcriptomic platforms such as GeoMx, Visium, and Xenium offer increasing spatial resolution to capture gene expression within an intact tissue architecture. Spatial transcriptomics has been applied to major pulmonary diseases—including lung cancer, pulmonary fibrosis, COPD, asthma, tuberculosis, and sarcoidosis—revealing localized, disease-specific transcriptional signatures that provide insights into their pathogenesis. These insights enable biomarker discovery, elucidation of drug resistance mechanisms, and the development of precision medicine strategies. Figure created with BioRender.com.

Schematic illustration of three widely utilized spatial transcriptomics platforms—GeoMx Digital Spatial Profiler (NanoString), Visium (10x Genomics), and Xenium (10x Genomics). GeoMx utilizes UV-induced photocleavage of oligonucleotide tags within predefined ROI, enabling spatial resolution of RNA or protein expression. Visium captures polyadenylated transcripts across tissue sections using spatially barcoded probes, offering moderate resolution suitable for transcriptome-wide profiling. Xenium utilizes iterative in situ hybridization and high-resolution imaging to achieve single-cell to subcellular transcriptomic mapping. Figure created with BioRender.com.

Above is a flow diagram providing a conceptual framework for selecting a spatial transcriptomics platform based on analytical goals and technical requirements. Xenium is optimal when high-resolution, single-cell, or subcellular transcript localization is required. Visium is suited for transcriptome-wide spatial profiling across whole tissue sections at moderate resolution. GeoMx is advantageous for targeted, multiplexed quantification of RNA and/or protein in user-defined regions. This decision tree facilitates methodological alignment between spatial resolution needs, molecular targets, and tissue compatibility in diverse biological and translational research contexts.

## 3. Published Data on Spatial Transcriptomics in Lung Cancer and Pulmonary Diseases

Recent advances in ST have enabled high-resolution mapping of cellular and molecular landscapes across a wide spectrum of pulmonary diseases. In lung cancer, ST has elucidated the spatial organization of tumor subpopulations, stromal architecture, and immune niches. In contrast, in fibrotic and obstructive lung conditions, ST has identified key cell types and signaling pathways that contribute to disease progression and tissue remodeling (Table 3).

### 3.1. Spatial Transcriptomics in Lung Cancer: Tumor Heterogeneity, Microenvironment, and Therapeutic Implications

ST platforms such as GeoMx DSP, Visium, and Xenium have been instrumental in elucidating the spatial dynamics between tumor cells and the tumor microenvironment. GeoMx has been applied in non-small cell lung cancer (NSCLC) to reveal region-specific expression patterns of immune checkpoint molecules, while Visium has enabled the delineation of heterogeneous tumor subtypes and their associated stromal compartments in breast cancer [5,29,35,52]. More recently, Xenium has provided single-molecule resolution of rare tumor subpopulations and their interactions with immune cells, uncovering mechanisms of immune evasion and therapeutic resistance. Collectively, these technologies have become pivotal tools in precision oncology, offering spatially resolved insights into cancer development, evolution, and response to therapy [35,50,53,54,55,56].

Among pulmonary diseases, lung cancer has been the most extensively studied using spatial transcriptomics, owing to its high intratumoral heterogeneity and clinical relevance. Spatial transcriptomics has provided novel insights into the spatial arrangement of immune inhibitory interactions during the histological progression of lung adenocarcinoma [57,58]. Luo et al. integrated single-cell RNA sequencing, spatial transcriptomics, and immunofluorescence to map co-inhibitory receptor–ligand pairs across lepidic, papillary, acinar, and solid histologic patterns. This study identifies several critical co-inhibitory receptor–ligand interactions, including PD1/PD-L1, PVR (poliovirus receptor)/TIGIT (T cell immunoreceptor with Ig and ITIM domains), and TIGIT/NECTIN2 (nectin cell adhesion molecule 2), which contribute to the recruitment of immunosuppressive cells, such as M2 macrophages and Tregs [7]. These interactions play a crucial role in creating an immunosuppressive microenvironment and inducing T cell exhaustion. This study highlights the molecular and cellular heterogeneity of lung adenocarcinoma (LUAD) and provides valuable insights for the development of novel immunotherapies targeting these immunosuppressive pathways [7]. Yan et al.’s study showed that spatial transcriptomics delineates molecular features and cellular plasticity in lung adenocarcinoma progression [48]. Integrative single-cell and spatial transcriptomics analysis reveals the Midkine (MDK)–Nucleolin (NCL) pathway’s role in shaping the immunosuppressive environment of lung adenocarcinoma [59].

Wang et al. investigate the molecular characteristics and cellular dynamics within different histologic subtypes of LUAD, including both indolent (lepidic) and aggressive subtypes such as micropapillary, solid, and poorly differentiated acinar [48]. The research combines spatial transcriptomics with multiplex immunohistochemistry to provide a comprehensive view of the transcriptional reprogramming and dynamic cell signaling that drive the progression of these subtypes. A key focus of the study is on the role of hypoxia-induced regulatory networks in facilitating the transition from indolent to aggressive subtypes. The findings revealed significant heterogeneity in the dedifferentiation states across the LUAD subtypes. This heterogeneity is a critical factor in the progression of the disease, as different subtypes exhibit varied degrees of dedifferentiation [48].

Choi et al. [49]. investigated the spatial organization and role of cancer-associated fibroblasts (CAFs) in lung squamous cell carcinoma (LUSC), a prevalent and aggressive type of lung cancer. Using ST, the study analyzes tissue samples from 33 LUSC patients to explore the spatial interactions between CAFs and tumor epithelium [49]. The findings show that the proximity of CAFs to tumor epithelial cells correlates with tumor size, metabolic activity, and recurrence-free survival. By characterizing CAFs based on their spatial relationships, the study identifies distinct molecular signatures tied to different fibroblast subpopulations. Additionally, barcode-based ST data from 8 LUSC patients reveal fibroblast regions with upregulated glycolysis pathways. This research highlights the importance of spatial dynamics within the tumor microenvironment and its implications for patient outcomes in LUSC [49]. Complementing these findings, Chao et al. [58]. reported a myofibroblastic CAF subpopulation marked by periostin (POSTN) expression, enriched in advanced NSCLC tumors. Spatial transcriptomics and multiplexed IHC revealed that POSTN+ CAFs colocalized with secreted phosphoprotein 1 (SPP1)+ macrophages and were associated with T cell exhaustion and reduced immune infiltration. These findings suggest that POSTN+ CAFs contribute to an immunosuppressive and invasive tumor microenvironment and are linked to poor prognosis [58].

One study investigates the diverse prognoses of early-stage LUAD patients with different histological subtypes. The simultaneous presence of multiple histological subtypes complicates clinical diagnosis and prognosis due to tumor complexity [50]. The research included 11 postoperative LUAD patients, all confirmed to be stage IA. scRNA-seq was performed on matched tumor and normal tissues, while three formalin-fixed, paraffin-embedded cases were analyzed using 10x Genomics Visium and DSP technologies. The analysis revealed distinct gene profiles for lepidic and acinar histological subtypes. ScRNA-seq results showed some concordance with clinicopathological findings. DSP proteomics and Visium transcriptomics identified a significant negative correlation (r = -0.886, *p* = 0.033) between the expression levels of CD8+ T cells and programmed cell death protein 1 (PD-L1) expression on tumor endothelial cells. Notably, the percentage of CD8+ T cells was lower in the acinar region compared to the lepidic region. These findings demonstrate the feasibility of assessing LUAD histological subtypes at the single-cell level and highlight the role of PD-L1-expressing tumor endothelial cells in suppressing immune-responsive CD8+ T cells in stage IA LUAD [50].

Zhang et al. analyzes primary and metastatic tumor specimens from 44 NSCLC) patients using spatial RNA sequencing to map the whole transcriptome of brain metastases (BrMs) [51]. The findings reveal extensive remodeling of the tumor microenvironment (TME) in the brain, including both the tumor immune microenvironment (TIME) and tumor brain microenvironment (TBME), creating an immunosuppressive and fibrogenic niche for BrMs. Key features of the brain TME include reduced antigen presentation, impaired B/T cell function, increased neutrophils and M2 macrophages, immature microglia, and reactive astrocytes. Gene expression analysis identifies immune regulation and fibrosis as major disrupted pathways in both the lung and brain TME. This study provides insights into lung cancer brain metastasis mechanisms, uncovers potential prognostic biomarkers, and suggests that treatments should be tailored to the immune and fibrotic status of BrMs [51]. Expanding on these findings, a more recent multimodal study profiled treatment-naive NSCLC brain metastases and matched primary tumors using single-nucleus RNA-seq, spatial transcriptomics, and whole-genome sequencing. This integrative analysis identified a chromosomal instability (CIN) high cancer cell subpopulation with neural-like transcriptional features, preexisting in primary tumors but enriched in brain metastases and spatially localized using matched single-cell resolution ST [60]. These results highlight the molecular and spatial adaptations of NSCLC cells during metastatic progression to the brain.

**Figure 4:**
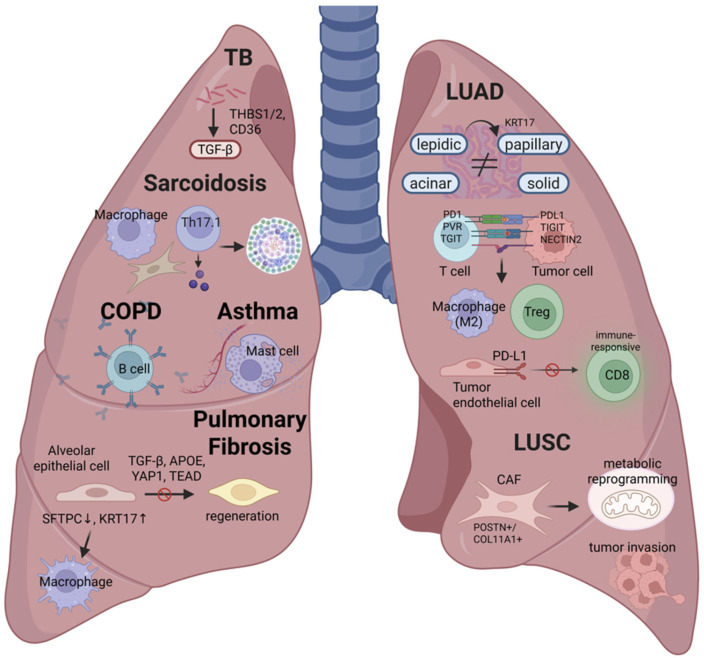
Spatial transcriptomic signatures across pulmonary diseases: cellular niches and molecular pathways.

ST has been applied to analyze the TIME of small cell lung cancer (SCLC), a neuroendocrine malignancy known for limited immune cell infiltration and poor responsiveness to immunotherapy. By integrating ultra-high-plex DSP with single-cell RNA sequencing, the authors generated a region-specific proteo-transcriptomic atlas from 44 treatment-naive SCLC tumors. This approach enabled the spatial resolution of over 18,000 transcripts and 60 immune-related proteins across histologically defined compartments—tumor nests, stromal regions, and adjacent non-tumor lung tissue. The spatial data revealed marked intra-tumoral heterogeneity in neuroendocrine features and immune infiltration, with NE-low subtypes showing greater infiltration by antigen-presenting myeloid cells and cytotoxic lymphocytes. Importantly, the study identified the Repressor Element 1 silencing transcription factor (REST) as a spatially localized marker of NE-low, immune-inflamed SCLC, correlating with enhanced antigen presentation and improved survival. By mapping cellular composition and transcriptional programs across spatial compartments, this work demonstrates the unique value of spatial transcriptomics in uncovering clinically relevant heterogeneity in SCLC—offering mechanistic insights into subtype-specific immune interactions and identifying REST as a potential biomarker for therapeutic stratification (Figure 4).

### 3.2. Spatial Transcriptomics in Non-Malignant Pulmonary Diseases: Inflammatory Niches and Fibrotic Remodeling

Beyond cancer, spatial transcriptomics have begun to illuminate the cellular and molecular architecture of non-malignant pulmonary diseases, including fibrotic and inflammatory lung disorders [61] (Figure 4). In a recent study, Vannan et al. applied sub-cellular resolution spatial transcriptomics to analyze the gene expression of over 1 million cells from 19 human lungs, comparing control and pulmonary fibrosis (PF) lungs [43]. The research identifies key cell types and molecular signatures that contribute to the pathogenesis of PF, a chronic lung disease characterized by progressive structural changes. By employing machine learning and trajectory analysis, the study tracks compositional and molecular changes in lung airspaces, starting with alveolar epithelial dysregulation and progressing to macrophage polarization. The findings reveal distinct molecularly defined spatial niches in both control and PF lungs, providing a comprehensive understanding of the heterogeneous disease mechanisms underlying PF. These insights, along with the analytical methods used, offer valuable approaches for future imaging-based spatial transcriptomic studies in other diseases [43]. Lovisa et al. utilized spatial transcriptomics to identify distinct fibrotic niches in the idiopathic pulmonary fibrosis (IPF) lung, which are characterized by aberrant alveolar epithelial cells within a microenvironment dominated by transforming growth factor-β (TGF-β) signaling, along with predicted upstream regulators such as TP53 and apolipoprotein E (APOE) [40].

While spatial transcriptomics has provided valuable insights into fibrotic remodeling in IPF, similar approaches have also been applied to COPD to explore localized immune structures and emphysematous changes. Using spatial transcriptomic and proteomic profiling of lung tissue from ever-smokers and COPD patients, Sauler et al. characterized lymphoid follicles (LFs) across varying degrees of emphysema [46]. They found that LFs in emphysematous lungs exhibited a distinct B cell–dominant transcriptional signature associated with chronic activation, inflammation, and antigen presentation, which contrasted with the anti-inflammatory profile seen in non-emphysematous COPD. These findings suggest that aberrant B cell activity within LFs may contribute to autoimmune-driven pathogenesis in emphysema.

Following the discussion on COPD, a recent study has applied spatial transcriptomics to investigate vascular alterations in early-life allergic asthma [62]. In a neonatal mouse model of house dust mite (HDM)-driven allergic asthma, mast cell (MC) activation was shown to disrupt endothelial–pericyte interactions in peribronchial adventitial regions, as MC proteases (e.g., tryptase) induced pericyte retraction and loss of the endothelial adhesion molecule N-cadherin, leading to focal pulmonary vascular remodeling [62]. Consistently, a spatial transcriptomic analysis of endobronchial biopsies from asthmatic children revealed that vessel-rich regions exhibit transcriptional signatures of intense vascular stress and remodeling alongside upregulated MC activation pathways, highlighting a spatially localized MC–pericyte axis driving early-life asthma-associated microvascular disruption [62].

Through spatial transcriptomics, granulomas in sarcoidosis were revealed to form within an immune-stimulatory environment composed of metabolically reprogrammed macrophages, cytokine-producing Th17.1 cells, and inflammatory fibroblasts, aberrantly repurposing gene regulatory programs associated with lymphoid organ development [44]. Regarding tuberculosis, spatial transcriptomics has enabled a detailed understanding of granuloma composition, spatial immune dynamics, and fibrosis-related gene expression, offering new insights into disease pathogenesis and potential therapeutic targets such as thrombospondin-1 (THBS1) [45].

Recent studies utilizing ST have identified discrete proinflammatory niches in the asthmatic airway wall, where atypical chemokine receptor 1 (ACKR1) mediates the local retention of immune mediators and amphiregulin-expressing mast cells are enriched. These findings highlight persistent tissue remodeling and spatially organized cellular interactions as promising therapeutic targets, even in the presence of anti-inflammatory treatment [47].

ST analysis has also been utilized to compare and characterize lung injury caused by SARS-CoV-2 and H1N1. While H1N1 showed strong antiviral immune activation, SARS-CoV-induced acute respiratory distress syndrome (ARDS) exhibited a fibroproliferative signature marked by epithelial-to-mesenchymal transition, vascular injury, and extracellular matrix remodeling. These distinct transcriptional profiles suggest virus-specific mechanisms of ARDS pathogenesis and highlight therapeutic targets for COVID-19.

Beyond malignancies, ST has emerged as a transformative tool in elucidating the pathophysiological mechanisms underlying non-malignant pulmonary diseases by uncovering region-specific immune and stromal alterations. Unlike conventional transcriptomic approaches, ST preserves tissue architecture, enabling the spatial mapping of gene expression to distinct cellular niches. This spatially resolved perspective facilitates the identification of localized immune cell aggregates, fibrotic remodeling zones, and aberrant epithelial–mesenchymal interactions across diseases such as IPF, COPD, granuloma, asthma, and ARDS. By reconstructing the tissue ecosystem in situ, ST provides mechanistic insights into disease initiation and progression—ranging from alveolar epithelial dysfunction to maladaptive immune responses—that are not discernible through bulk or single-cell RNA sequencing alone. These capabilities position ST as a powerful approach for uncovering fundamental drivers of chronic inflammation and tissue remodeling, ultimately paving the way for spatially informed diagnostic frameworks and targeted therapeutic strategies (Table 3).

This figure illustrates spatial-transcriptomics-based findings across major pulmonary diseases, highlighting key cellular and molecular features identified in lung tissue. In tuberculosis (TB), M. tuberculosis infection activates TGF-β signaling through the upregulation of THBS1/2 and CD36. Sarcoidosis is characterized by granuloma formation involving Th17.1 cells, reprogrammed macrophages, and remodeling fibroblasts. In COPD, centrilobular emphysema regions show enrichment of genes related to B cell maturation and antibody production. Asthma displays vascular-rich regions with signs of vascular stress, remodeling, and mast cell activation. In pulmonary fibrosis, alveolar epithelial dysregulation (SFTPC↓, KRT17↑) and macrophage polarization (SPP1, CHI3L1) are observed along with impaired regeneration driven by altered signaling (TGF-β, APOE, YAP1, TEAD). In LUAD, spatial transcriptomics has identified distinct histologic patterns (lepidic, papillary, acinar, solid) associated with key co-inhibitory receptor–ligand interactions, such as PD1–PDL1, PVR–TIGIT, and NECTIN2–TIGIT, which promote recruitment of M2 macrophages and Tregs. Dedifferentiation from lepidic to micropapillary subtypes is linked to KRT17 overexpression and macrophage spatial heterogeneity, while PD-L1+ tumor endothelial cells in early-stage LUAD suppress immune reactive CD8+ T cell responses. LUSC demonstrates spatially distinct CAF populations (POSTN+, COL11A1+) linked to tumor invasion and metabolic reprogramming. These findings reveal how spatial transcriptomics uncovers disease-specific microenvironments, providing new insights into the pathogenesis of various pulmonary disorders.

## 4. Ongoing Clinical Trials Utilizing Spatial Transcriptomics in Lung Cancer and Pulmonary Diseases

ST has increasingly attracted interest as a biomarker discovery and stratification tool in clinical settings. The following clinical trials investigate the use of ST in various pulmonary disease contexts, along with the platforms being employed and preliminary insights, where available (Table 4).

The open-label, randomized, single-center translational study (NCT06893354) aims to investigate the tumor microenvironment remodeling following lorlatinib induction therapy in surgically resectable ALK-positive NSCLC. The study utilizes scRNA-seq and ST to identify the mechanisms of disease persistence and potential primary resistance to lorlatinib, which is used as an induction therapy in ALK-rearranged NSCLC. The findings from this study are expected to provide insights into the primary resistance mechanisms, thereby improving our understanding of how tumor microenvironment dynamics contribute to treatment outcomes in ALK-positive NSCLC.

The NCT04789252 trial investigates the functional heterogeneity and spatial distribution of dendritic cell (DC) subtypes and other myeloid cells in the tumor microenvironment of NSCLC using single-cell RNA sequencing and spatial transcriptomics. By integrating spatial data with survival outcomes, the study aims to identify immune cell signatures associated with response or resistance to immunotherapy. Another exploratory single-arm study (NCT06987734) in resectable stage II–IIIA NSCLC integrates spatial transcriptomics and single-cell sequencing to investigate immune microenvironment changes before and after perioperative PD-L1 monoclonal antibody with Sugemalimab treatment. A spatial analysis of tumor tissues pre- and post-treatment aims to reveal mechanisms of response and resistance to immunotherapy. A retrospective cohort study (NCT05055947) in extensive-stage small-cell lung cancer utilizes spatial transcriptomics, RNA-seq, WES, and multi-omics analyses to identify biomarkers predicting response to first-line Atezolizumab plus platinum-etoposide therapy. Although focused on SCLC, findings may offer insights into immune-related spatial signatures relevant to NSCLC immunotherapy strategies.

Another clinical trial (NCT06396910) compares the immune microenvironments of granulomas in tuberculosis and sarcoidosis, two granulomatous diseases, using in situ sequencing (ISS) and multiplexed immunolabelling of tissue sections. The research aims to understand how immune molecule localization within granulomas influences disease progression in TB and sarcoidosis. It investigates differences in immune landscapes between TB granulomas with or without necrosis, acute versus chronic sarcoidosis, and histologically similar granulomas in both diseases. By mapping 65 immune markers in lung tissue, the study correlates immune responses with granuloma histology, bacterial content in TB, and disease type. The findings offer new insights into disease mechanisms, providing potential targets for therapy and improving diagnostic approaches for TB and sarcoidosis. This research underscores the value of spatial transcriptomics in understanding complex immune responses in granulomatous diseases.

## 5. Future Perspectives and Clinical Significance

### 5.1. Spatial Transcriptomics and Multomics Integration

ST holds immense promise for advancing both basic and translational research in lung cancer and other pulmonary diseases, owing to its unique ability to preserve tissue architecture and gene expression patterns, thereby providing unparalleled spatial insights. One major growth area is integration with other omics, such as proteomics or metabolomics, where multi-omics approaches can illuminate crosstalk among protein modifications, metabolic pathways, and transcriptomic signals [14,35,63]. These comprehensive datasets could expedite biomarker discovery and aid in identifying novel therapeutic targets. Recent innovations in ST and multi-omics integration have significantly advanced our understanding of tissue architecture and cellular dynamics. Novel computational tools, such as Spatial Integration of Multi-Omics (SIMO), Multi-Omics Imaging Integration Toolset (MIIT), and dual-path graph attention auto-encoder (SSGATE), have enabled the alignment of ST data with single-cell RNA-seq, Assay for Transposase-Accessible Chromatin with sequencing (ATAC-seq), DNA methylation, and proteomic profiles, thereby enhancing the resolution of spatial heterogeneity and cellular interactions in complex tissues [64,65,66]. High-throughput platforms like GeoMx DSP, CosMx Spatial Molecular Imager (SMI), and Spatial Multi-Omics (SM-Omics) facilitate multiplexed RNA and protein analysis at regional to single-cell scales, revealing spatially resolved biomarkers and immune niches in diseases such as lung fibrosis, cancer, and colorectal inflammation. These integrative approaches are redefining tissue biology by linking molecular data with spatial context, despite ongoing challenges in data harmonization and computational scalability.

Another key trend is the drive toward higher resolution and throughput: emerging chemistries, refined microfluidic techniques, and cutting-edge microscopy are propelling ST toward single-cell or even subcellular resolution. Such granular data will be critical for detecting rare or transient cell types and pinpointing subcellular expression shifts involved in disease progression [63].

### 5.2. Cutting-Edge Advances in Spatial Transcriptomics Methodologies

Recent advancements in ST have led to the development of diverse technical platforms, each with unique strengths and limitations. Broadly, these can be categorized into three major methodological classes. Laser Capture Microdissection (LCM)-based approaches enable region-specific transcriptomic profiling by physically isolating defined areas of tissue under microscopic guidance, thereby preserving spatial resolution while allowing downstream sequencing [67]. In Situ Imaging-based methodologies, such as MERFISH (Multiplexed Error-Robust Fluorescence In Situ Hybridization) (Vizgen, Cambridge, MA, USA) and seqFISH (sequential FISH), use multiplexed fluorescence imaging to generate high-resolution spatial maps of RNA molecules within intact tissues, often at single-molecule resolution [68,69]. Spatial Indexing-based techniques, including barcoded bead arrays like 10x Visium and Slide-seq, assign transcriptomic reads to spatial locations by capturing mRNA on predefined barcoded coordinates embedded within the slide. These innovations have significantly expanded the spatial resolution, molecular throughput, and multimodal integration capabilities of ST, enabling deeper insights into complex biological systems such as the tumor microenvironment and fibrotic lung remodeling.

### 5.3. Spatial Transcriptomics and Artificial Intelligence

Recently, new machine learning and artificial intelligence algorithms are being tailored to process increasingly large and complex spatial data, revealing subtle spatial correlations, predictive gene signatures, and intricate cell–cell communication networks that may be overlooked by standard statistical methods [70]. Recent advances in probe design and sample processing have also improved compatibility with FFPE tissues, broadening the clinical applicability of ST across retrospective cohorts and routine diagnostic workflows. Finally, the potential for clinical diagnostic applications is rapidly expanding, with ST-based assays poised to refine disease classifications and predict treatment responses more accurately. By integrating ST data into pathology workflows, clinical teams could gain unprecedented spatial and molecular context for each patient’s disease, taking traditional histopathology into the next generation of digital and molecular pathology—ultimately enabling spatially informed precision medicine [37,71,72,73].

## 6. Challenges and Considerations

Several key considerations remain before ST can be integrated into routine clinical practice. Cost and accessibility are primary concerns, as the current expense of ST analyses remains prohibitive for large-scale patient cohorts. However, ongoing advancements in technology and increased market competition are expected to progressively lower these financial barriers. Equally critical is the need for methodological standardization. Variability in protocols for tissue processing, data acquisition, and computational workflows highlights the necessity for harmonized procedures and universally accepted quality metrics to ensure reproducibility and clinical applicability.

Another crucial challenge lies in the ethical and regulatory landscape. As with other high-resolution genomic approaches, ST generates highly sensitive and identifiable molecular data, necessitating rigorous safeguards for patient privacy and stringent data governance frameworks [6,36].

Importantly, the intrinsic heterogeneity within tissues—particularly in tumors and many inflammatory or fibrotic diseases—underscores the significance of region selection during spatial transcriptomic analysis. Accurate identification of ROIs is essential for ensuring biologically meaningful and clinically relevant interpretations. This process demands a deep understanding of disease pathology, clearly defined analytical objectives, and close interdisciplinary collaboration with expert pathologists. Integrating pathological expertise into spatial study design enhances precision, minimizes bias, and ensures that the spatially resolved data are contextually anchored within the histological and clinical landscape of the disease. Collectively, addressing these technical, logistical, and conceptual challenges is vital for the responsible and effective integration of ST into precision medicine frameworks.

## 7. Conclusions

ST represents a transformative leap in our ability to study lung cancer and pulmonary diseases. By preserving the spatial context of gene expression, it offers unparalleled insights into tumor microenvironments, tissue architecture, and complex cell–cell interactions underpinning disease pathology. The ongoing evolution of ST platforms—exemplified by GeoMx, Xenium, and Visium—has expanded both the analytical scope and spatial resolution of this field. Current and future clinical trials integrating ST underscore the technology’s potential to advance precision medicine by enabling biomarker discovery and spatially guided therapeutic stratification.

The continued advancement of ST methods, especially in synergy with other spatial omics technologies, will refine our understanding of lung pathophysiology. Ultimately, as costs decrease and protocols become standardized, spatial transcriptomics is expected to become an integral part of both research laboratories and clinical diagnostic workflows, fundamentally reshaping how we diagnose, prognosticate, and treat lung cancer and a broad range of pulmonary diseases.

ST, by preserving spatial context, has significantly advanced our understanding of both the structural and functional organization of normal tissues and the pathophysiology of various diseases—particularly cancer and the TME—in ways that single-cell RNA sequencing alone could not achieve. ST enables high-resolution mapping of gene expression across the tissue architecture, offering critical insights into transitional zones, intratumoral heterogeneity, and the dynamic interactions among immune, stromal, and malignant cells within the TME. These insights have facilitated the identification of predictive biomarkers for immunotherapy response and the discovery of novel therapeutic targets. Despite its transformative potential, ST still faces several limitations in clinical and research applications. High costs and the restricted size of the ROIs can limit its scalability and raise concerns about whether the sampled regions accurately represent the overall tumor landscape. Addressing these challenges through technological innovation and improved spatial coverage will be essential for broader adoption and clinical translation.

## Figures and Tables

**Table 1:** Comparative overview of WES, WGS, bulk RNA-seq, single-cell RNA-seq, and spatial transcriptomics.

Feature	WES	WGS	Bulk RNA-seq	scRNA-seq	Spatial Transcriptomics
Data Type	DNA (exons only)	DNA (whole genome)	RNA (pooled sample)	RNA (individual cells)	RNA in intact tissue (spots or single cells)
Coverage	Protein-coding regions (~1–2% of genome)	Entire genome	Transcriptome (averaged)	Transcriptome (per cell)	Whole/partial transcriptome; spatial resolution varies by platform (spot to subcellular)
Resolution	Detects exon variants only	Detects all variants (SNVs, SVs, etc.)	Average expression across mixed cells	Expression profiles at single-cell level	Multi-/single-cell gene expression + tissue context
Applications	Disease-causing coding variants, driver mutations	Comprehensive variant analysis, structural variation	Differential expression, biomarker discovery	Heterogeneity, rare cell types, lineage tracing	Cell–cell interaction, tissue microenvironment, histology integration
Complexity	Moderate; targeted capture + NGS	High; large data, complex variant calling	Moderate; standard RNA-seq pipeline	High; single-cell isolation, large datasets	High; specialized platforms, data integration (image + transcriptome)
Typical Cost	Medium (less than WGS)	Highest among options	Lower (per sample)	Higher (per sample), specialized library prep	High (instrumentation, reagents, large data)
Key Limitations	Misses noncoding variants, no expression data	Expensive, high storage/computing needs	Loses single-cell detail, masks rare populations	No spatial info, can alter cell states upon dissociation	Technology still evolving, fewer high-throughput options, costlier

Abbreviation: WES: whole exome sequencing, WGS: whole genome sequencing, RNA-seq: RNA sequencing, scRNA-seq: single-cell RNA sequencing, spatial transcriptomics: spatially resolved transcriptomics, SNVs: single nucleotide variants, SVs: structural variants, NGS: next-generation sequencing, UTRs: untranslated regions.

**Table 2:** Comparison of leading spatial transcriptomics platforms.

Platform	GeoMx (NanoString)	Xenium (10x Genomics)	Visium (10x Genomics)
Technical Principle	Photocleavable oligo tags bound to probes or antibodies; UV-based ROI illumination	In situ hybridization of barcoded probes for single-molecule detection	Spatially barcoded capture spots on slides (oligo-dT or targeted probes)
Resolution	Multi-cell to near single-cell (depends on ROI size)	Single-molecule or single-cell resolution	Multi-cell per spot (55–100 µm diameter)
Molecular Targets	RNA (up to 18,000-plex) or proteins (100+ markers)	RNA transcripts (single-molecule sensitivity)	Poly(A)+ RNA; a targeted probe approach available for FFPE
Sample Compatibility	FFPE or fresh/frozen	Primarily fresh/frozen; must be compatible with in situ protocols	Fresh/frozen standard; FFPE version uses targeted capture
Key Strengths	- High multiplexing (RNA and protein) - Flexible ROI selection - Straightforward FFPE use	- Subcellular resolution - Direct detection of transcripts - Seamless integration with 10x single-cell data	- Relatively user-friendly workflows - High throughput - Well-established pipelines - Lower cost than single-molecule approaches
Key Limitations	- Typically not single-cell resolution by default - Specialized laser hardware - Selection bias due to ROI-based analysis	- Higher cost and technical demands - Risk of tissue damage during in situ hybridization - Potential autofluorescence issues	- Spots cover multiple cells (limited resolution) - Mostly poly(A)+ transcripts only - FFPE version is targeted, not whole-transcriptome
Use Cases	- Targeted studies of specific regions - Protein + RNA multiplexing - Archival/clinical FFPE samples	- High-resolution mapping of gene expression - Small tissue areas or subcellular studies - Integration with scRNA-seq - Target validation	- Moderate resolution for broad tissue survey - Cost-effective screening - Established pipelines for large-scale projects - Target discovery or screening

Abbreviations: FFPE, formalin-fixed paraffin-embedded; ROI, region of interest; scRNA-seq, single-cell RNA sequencing.

**Table 3:** Representative spatial transcriptomics studies in lung cancer and pulmonary diseases.

Diseases	Key Findings	ST Platforms	Sample Size	Reference
Pulmonary Fibrosis (PF)	Mapped alveolar epithelial dysregulation (SFTPC↓, KRT17↑) and macrophage polarization (SPP1/CHI3L1) across remodeling gradients in idiopathic PF.	Xenium, Visium	35 unique lungs	[43]
Pulmonary Fibrosis,	IPF lungs show arrested alveolar cell regeneration, unlike the active repair seen in the BLM model, due to differences in signaling molecules (TGF-β, APOE, YAP1, TEAD) and immune cell profiles.	Visium	4 Human IPF, 6 bleomycin-induced mouse lungs	[40]
Sarcoidosis	Metabolically reprogrammed macrophages, cytokine-producing Th17.1 cells, and fibroblasts with inflammatory and tissue-remodeling phenotypes are key players in granuloma formation	Visium	12 patients	[44]
Tuberculosis	Mtb infection can activate TGF-β signaling by inducing the expression of THBS1/2 and CD36	Visium	2 patients, 2 controls	[45]
COPD, emphysema	The extent of centrilobular emphysema was significantly associated with genes involved in B cell maturation and antibody production.	GeoMx DSP	40 patients	[46]
Asthma	The asthma airway mucosa exhibited a distinct remodeling program within these cellular ecosystems, marked by increased proximity between key cell types	Xenium, GeoMx DSP	20 patients, 8 controls	[47]
Lung Adenocarcinoma (LUAD)	Identified co-inhibitory ligand-receptor interactions (NECTIN2/TIGIT, PVR/TIGIT) in solid histological patterns, correlating with poor immunotherapy response.	Visium	2 tumor samples	[7]
LUAD Progression	Linked dedifferentiation trajectories (lepidic → micropapillary) to KRT17 overexpression and macrophage spatial heterogeneity.	Visium	10 patients	[48]
Lung Squamous Cell Carcinoma (LUSC)	Revealed spatially distinct CAF subtypes (POSTN+/COL11A1+) driving tumor invasion and metabolic reprogramming (HK2/LDHA).	MERFISH, Visium	33 patients	[49]
LUAD Histologic Subtypes	Tumor endothelial cells that express PD-L1 in stage IA LUAD suppress immune-responsive CD8+ T cells.	Visium	11 postoperative LUAD patients	[50]
NSCLC Brain Metastasis	Characterized transcriptomic divergence between primary tumor cores (PanCK+) and brain TIME/TBMEs.	GeoMx DSP	44 patients	[51]

Abbreviations: PF, pulmonary fibrosis; IPF, idiopathic pulmonary fibrosis; COPD, chronic obstructive pulmonary disease; LUAD, lung adenocarcinoma; LUSC, lung squamous cell carcinoma; NSCLC, non-small cell lung cancer; ST, spatial transcriptomics; TTI: transcriptional target intersection; MERFISH: multiplexed error-robust fluorescence in situ hybridization; DSP: digital spatial profiler; TIME: tumor immune microenvironment; TBME: tumor brain microenvironment.

**Table 4:** On ongoing clinical trials using ST in pulmonary lung diseases.

Trial ID	Phase	Focus	Disease
NCT06893354	4	Explore the Mechanisms Underlying Disease Resistance and Potential Primary Resistance Mechanism	ALK (+) NSCLC
NCT04789252	Observational	Heterogeneity of Dendritic Cells in NSCLC	NSCLC
NCT06987734	2	ST explores the changes in the iTME before and after suglizumab administration	NSCLC
NCT05055947	Observational	ST related biomarkers to predict the efficacy of Atezolizumab plus etoposide and platinium	SCLC [ES]
NCT06396910	NA	Immunological micro-environments of granulomas	Tuberculosis and sarcoidosis

NA: non-applicable, ST, spatial transcriptomics; NSCLC, non-small cell lung cancer; SCLC [ES], extensive stage small cell lung cancer; ALK, anaplastic lymphoma kinase; iTME, immune tumor microenvironment; Atezolizumab, anti–PD-L1 immune checkpoint inhibitor; Sugemalimab, anti–PD-L1 immune checkpoint inhibitor.

## Abbreviations

ALK: Anaplastic Lymphoma Kinase, ARDS: Acute Respiratory Distress Syndrome, ATAC-seq: Assay for Transposase-Accessible Chromatin using sequencing, CAF: Cancer-Associated Fibroblast, CIN: Chromosomal Instability, COPD: Chronic Obstructive Pulmonary Disease, CSF1R: Colony Stimulating Factor 1 Receptor, CT: Computed Tomography, DSP: Digital Spatial Profiler, FFPE: Formalin-Fixed Paraffin-Embedded, GEO: Gene Expression Omnibus, HDM: House Dust Mite, H&E: Hematoxylin and Eosin, ICI: Immune Checkpoint Inhibitor, IPF: Idiopathic Pulmonary Fibrosis, ISS: In Situ Sequencing, LF: Lymphoid Follicle, LUAD: Lung Adenocarcinoma, LUSC: Lung Squamous Cell Carcinoma, MC: Mast Cell, mTLS: Mature Tertiary Lymphoid Structure, mRNA: Messenger Ribonucleic Acid, MSI: Mass Spectrometry Imaging, NECTIN2: Nectin Cell Adhesion Molecule 2, NGS: Next-Generation Sequencing, NSCLC: Non-Small Cell Lung Cancer, PD-L1: Programmed Death-Ligand 1, PET: Positron Emission Tomography, POSTN: Periostin, REST: RE1 Silencing Factor, ROI: Region of Interest, RNA: Ribonucleic Acid, RNA-seq: RNA Sequencing, scRNA-seq: Single-Cell RNA Sequencing, SIMO: Spatial Integration of Multi-Omics, SCLC: Small Cell Lung Cancer, SPP1: Secreted Phosphoprotein 1, ST: Spatial Transcriptomics, TBME: Tumor Brain Microenvironment, TGF-β: Transforming Growth Factor Beta, TIGIT: T cell immunoreceptor with Ig and ITIM domains, TIME: Tumor Immune Microenvironment, TME: Tumor Microenvironment, Treg: Regulatory T Cell, WES: Whole Exome Sequencing, WGS: Whole Genome Sequencing, YAP1: Yes-Associated Protein 1.
